# Bird trematodes in the era of rapid environmental change

**DOI:** 10.1186/s13071-026-07470-6

**Published:** 2026-06-03

**Authors:** Petr Heneberg

**Affiliations:** Charles University, Third Faculty of Medicine, Ruská 87, CZ-100 00 Prague, Czech Republic

**Keywords:** Avian schistosome dermatitis, Cercarial emergence phenology, Cryptic species complexes, Functional homogenization, Host switching via ecological fitting, Temperature-dependent cercarial production

## Abstract

**Background:**

Rapid climate warming, hydrological regulation, eutrophication, pollution, and habitat conversion are reshaping aquatic ecosystems in which bird trematodes complete complex life cycles. Because birds act as definitive hosts, dispersal agents, and trophic links among habitats, we provide a bird-focused review aimed at improving our understanding of how environmental change affects trematode transmission, community structure, and biogeography.

**Methods:**

We reviewed evidence on avian trematodes across freshwater, coastal, artificial wetland, agricultural, and high-latitude systems. The review integrates mechanistic studies of temperature-dependent intramolluscan development, cercarial emergence, survival, and infectivity with field studies documenting changes in hydrology, intermediate-host availability, bird phenology, habitat use, and trematode community composition.

**Results:**

Environmental change affects bird trematodes through multiple interacting pathways. Warming can accelerate cercarial production and shift emergence phenology, but effects are nonlinear and stage-specific because higher temperatures may also reduce cercarial survival or disrupt synchrony with bird hosts. Hydrological alteration, eutrophication, and pollution alter snail and second intermediate host communities, creating spatially heterogeneous transmission mosaics. Changes in bird distribution, migration timing, diet, and use of artificial habitats can either maintain transmission, increase exposure to generalist trematodes, or interrupt complex life cycles. Long-term and molecular studies indicate that disturbed systems may lose specialist trematodes, become functionally homogenized, or conceal cryptic lineage turnover under apparently stable morphology-based species lists.

**Conclusions:**

Bird–trematode systems respond to environmental change in context-dependent ways rather than through uniform increases or decreases in transmission. Birds can amplify, disperse, buffer, or interrupt trematode life cycles depending on host competence, movement, habitat connectivity, and intermediate-host availability. Future surveillance should combine stage-specific parasite sampling, molecular identification, bird movement data, and remote sensing to improve forecasts of trematode redistribution and transmission risk under ongoing environmental change.

**Graphical Abstract:**

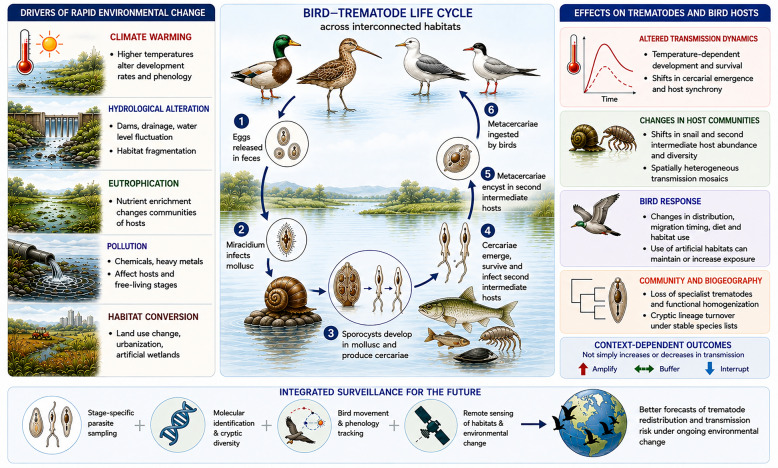

The graphical abstract was prepared by the authors with the assistance of generative artificial intelligence tools for illustrative visual elements and layout development. The authors subsequently edited, assembled, reviewed, and approved the final graphical abstract and take full responsibility for the accuracy of its scientific content.

## Background

Trematodes are digenetic parasitic flatworms with complex life cycles that typically involve a first intermediate molluscan host, one or more secondary intermediate hosts, and a definitive vertebrate host. Completion of these cycles depends on successful transmission across multiple host species and on free-living stages directly exposed to environmental conditions. Even small changes in temperature, water availability, or habitat structure can alter larval development, emergence, and survival, although responses vary among taxa and ecosystems [1, 2]. Freshwater and coastal systems have shown rapid shifts in trematode prevalence and intensity after environmental disturbance, indicating high sensitivity to ecological change [3, 4].

Birds are definitive hosts for a broad spectrum of trematode species, especially in aquatic and semiaquatic ecosystems. Waterfowl, shorebirds, and seabirds are consistently associated with high trematode richness and frequent mixed infections, reflecting repeated exposure over time [5, 6]. Many bird species accumulate trematode infections across multiple breeding and wintering seasons, consistent with their relatively long lifespans and repeated contact with infected intermediate hosts [7, 8]. Migratory birds also transport trematodes across large spatial scales among wetlands, river basins, and coastal habitats [9, 10]. By moving among feeding, roosting, and breeding sites, birds link aquatic parasite transmission to terrestrial and aerial landscape components [11, 12]. Experimental exclusion of bird hosts reduced trematode prevalence in intermediate hosts, confirming their functional role in maintaining parasite populations [8, 13]. Thus, birds are active drivers of trematode persistence rather than incidental final hosts.

Despite extensive research on bird trematodes, reviews that place birds at the center of trematode ecology remain limited. Reviews of trematode biology often emphasize molluscan hosts, larval ecology, or medically and veterinary important systems, with avian hosts treated mainly in taxonomic or regional contexts [14, 15]. Conversely, reviews of avian parasitology have mainly focused on blood parasites, gastrointestinal nematodes, and ectoparasites, with limited integration of trematodes into broader avian ecological analyses [16, 17]. As a result, patterns emerging from studies of bird trematodes remain fragmented across ecosystems and disciplines [18].

We argue that a bird-focused review is needed to interpret trematode responses to rapid environmental change. We integrate research on avian trematodes in relation to climate change, land-use change, hydrological alteration, and shifting species distributions, with explicit emphasis on avian life-history traits and movement ecology. By synthesizing evidence across freshwater, marine, and coastal systems, we aim to clarify how environmental change reshapes trematode transmission, community composition, and biogeography in avian hosts.

## Environmental change as a multilayered stressor of trematode life cycles

### Climate warming and thermal sensitivity

Temperature is a primary abiotic driver of trematode development, transmission, and survival across life-cycle stages [1, 15]. It regulates intramolluscan development, cercarial production, and the timing of emergence in many species [19, 20]. Higher water temperatures can shorten prepatent periods in snail hosts, leading to earlier emergence and higher daily cercarial output under both laboratory and field conditions [21, 22]. Seasonal and interannual temperature variation can explain much of the fluctuation in cercarial emergence and infection pressure on downstream hosts, including birds [23, 24].

**Fig. 1:**
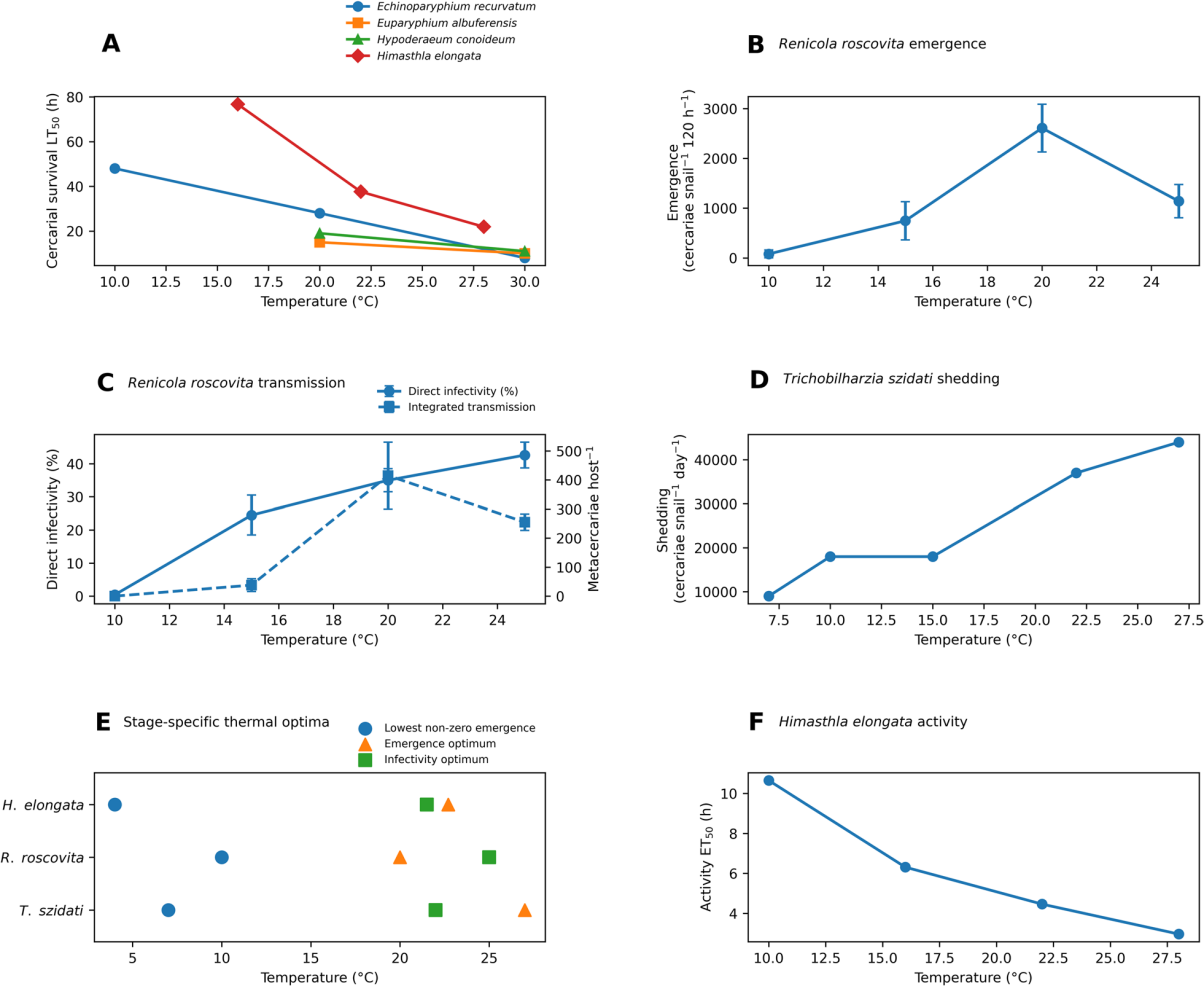
Temperature acts on multiple, partially decoupled stages of bird trematode life cycles, generating nonlinear and species-specific constraints on survival, propagule supply, and realized transmission that cannot be inferred from any single trait in isolation. **A** Temperature-dependent cercarial survival expressed as LT_50_. Points show the time at which 50% of cercariae were no longer alive or active under each experimental temperature. Data from McCarthy [166], Toledo et al. [168], and Díaz-Morales et al. [162]. **B** Temperature dependence of cercarial emergence in a marine bird trematode. The mean emergence of *Renicola roscovita* cercariae from the first intermediate host is shown across the four experimental temperatures (10, 15, 20, and 25 °C). Points represent the mean number of cercariae released per snail over a 120 h period, with error bars indicating SDs. Emergence exhibited a unimodal response, with maximal cercarial output at 20 °C and reduced emergence at both lower and higher temperatures. This pattern identifies a narrow thermal window that maximizes propagule supply to second intermediate hosts and, indirectly, to definitive bird hosts. Data from Thieltges and Rick [23]. **C** Temperature dependence of transmission success to second intermediate hosts. The effects of temperature on the transmission success of the marine bird trematode *Renicola roscovita* are shown using two complementary metrics. Circles and solid lines represent direct infectivity of cercariae to the second intermediate host (*Cerastoderma edule*), expressed as the percentage of hosts infected following standardized exposure. Squares and dashed lines represent integrated transmission success measured in mesocosm experiments, expressed as the mean number of metacercariae per host. The error bars indicate SDs. Transmission success increases from cooler to moderate temperatures, reaching a maximum at 20–25 °C, suggesting that realized transmission reflects the combined effects of emergence, survival, and infectivity rather than emergence alone. The data were adapted from Thieltges and Rick [23]. **D** Temperature dependence of cercarial shedding intensity in *Trichobilharzia szidati*. Mean daily cercarial shedding from *Lymnaea stagnalis* is shown across five experimental temperatures (7, 10, 15, 22, and 27 °C), expressed as cercariae snail^−^1 day^−^1. Shedding increased strongly with temperature, from 9000 cercariae snail^−^1 day^−^1 at 7 °C to 44,000 cercariae snail^−^1 day^−^1 at 27 °C, indicating a marked increase in propagule supply under warmer conditions. Data from Al Jubury et al. [161]. **E** Stage-specific thermal optima and lower thresholds across selected bird trematodes. This panel summarizes, for *T. szidati*, *R. roscovita*, and *Himasthla elongata*, the lowest non-zero emergence or shedding temperatures, emergence optima or peak emergence, infectivity optima or highest infectivity, and integrated transmission optima. The panel highlights that different life-cycle stages often show distinct thermal thresholds and optima, emphasizing partial decoupling among propagule supply, host infection, and realized transmission. Data summarized from Al Jubury et al. [161], Thieltges and Rick [23], and Díaz-Morales et al. [162]. **F** Temperature dependence of cercarial activity in *H. elongata*. Cercarial activity is expressed as ET_50_ [h], the time at which 50% of cercariae ceased active swimming. Activity ET_50_ declined from 10.65 h at 10 °C to 2.96 h at 28 °C, indicating progressively shorter activity periods with increasing temperature. Data from Díaz-Morales et al. [162]

Temperature also directly affects cercarial longevity, activity, and infectivity in the external environment (Fig. 1). Higher temperatures generally increase swimming activity and host-encounter rates but shorten survival, creating a trade-off between transmission opportunity and lifespan [1, 25]. Controlled infection experiments show that infectivity in second intermediate hosts may peak at intermediate temperatures and decline sharply at higher temperatures because of rapid energy depletion or thermal stress [26, 27] (Fig. 1). These shifts in cercarial performance directly influence the probability that birds acquire infections via trophic or direct transmission routes [8].

Thermal responses differ among bird-infecting trematode taxa (Fig. 1). Optimal temperatures for cercarial emergence, survival, and infectivity vary by species, reflecting differences in physiology and evolutionary history [20, 24]. Some temperate freshwater species show reduced cercarial emergence or survival outside moderate temperature ranges [27, 28], whereas species from shallow or thermally variable habitats may maintain emergence across wider temperature ranges or at higher temperatures [26, 29]. Warming can also shift trematode assemblage composition toward species with faster development and higher thermal tolerance [30, 31].

Temperature effects on transmission are often nonlinear. Cercarial production and infectivity can decline abruptly beyond upper thermal limits, even in species that benefit from moderate warming [20, 22]. Extreme temperatures can suppress emergence, increase larval mortality, and disrupt synchronization between parasite release and host availability [25, 27]. Because thermal sensitivity differs among life-cycle stages, stage-specific bottlenecks may constrain overall transmission despite improved performance elsewhere [15, 24]. Climate change is therefore likely to produce heterogeneous, context-dependent effects on trematode transmission to birds rather than uniformly increasing infection pressure.

### Altered hydrology and water availability

Hydrological regimes shape trematode transmission by regulating habitat availability, host density, and contact rates among hosts and free-living stages [11, 12]. Changes in precipitation patterns, including more frequent droughts and floods, alter the abundance and spatial distribution of intermediate snail hosts, which are essential to trematode life cycles [33, 34]. Prolonged drought can reduce snail populations and local trematode persistence by eliminating suitable habitats and interrupting transmission [29, 35]. In contrast, flooding can increase habitat connectivity, promote dispersal of snails and cercariae, and trigger short-term increases in transmission intensity [36–39].

River regulation (dams, abstraction, and channel modification) also alters flow velocity, sedimentation, and temperature regimes, with direct consequences for snail communities and trematode transmission [40, 41]. Reduced flow and stabilized water levels downstream of dams may favor suitable snail hosts and increase local trematode prevalence [35, 42]. Conversely, high-flow releases and artificial flow variability can reduce snail densities and suppress transmission by displacing hosts and larval stages [34, 43]. Hydrological regulation is thus a significant source of spatial heterogeneity in the infection pressure exerted by trematodes on birds using regulated waterways.

Snail hosts respond rapidly to changes in water availability, making transmission highly sensitive to hydrological timing and duration. Field experiments and long-term observations show that seasonal drying and reflooding constrain the period during which snails are infected and produce cercariae [36, 44]. Shortened hydroperiods can reduce cumulative cercarial output by limiting intramolluscan development time, even when snails persist [29, 34]. Such shifts can decouple parasite emergence from periods of peak bird abundance, reducing infection success in definitive hosts [8].

Ephemeral wetlands are extreme cases of hydrological variability and are becoming more common under climate and land-use change. In temporary ponds and seasonal marshes, trematode communities often show rapid turnover, low richness, and dominance by species with rapid development and high cercarial output [26, 45]. Birds using these habitats as stopover or breeding sites may experience highly variable trematode exposure depending on hydroperiod length and inundation timing [9, 45]. This likely shifts transmission toward temporally compressed, spatially heterogeneous dynamics, with unpredictable effects on infection risk in birds. This pattern does not apply equally to wetlands that expand seasonally during regular floods but retain refugia for snails and fish during the rest of the year. Such wetlands can support highly diverse, species-rich trematode communities, and receding waters may annually increase bird access to intermediate hosts, as documented for the former oxbow lake Pansee in Central Europe (J. Sitko, unpubl.).

### Eutrophication, pollution, and chemical stressors

Nutrient enrichment from agricultural runoff, sewage discharge, and urbanization is widely known to alter aquatic community structure and parasite transmission dynamics [46, 47]. Eutrophication often increases the abundance and biomass of intermediate snail hosts by boosting primary productivity and food availability [36, 48]. Experimental manipulations show that elevated nutrient levels can drive rapid snail population growth and increase trematode prevalence within snail hosts [47, 50]. Higher snail densities can then increase ecosystem-level cercarial output and exposure risk for second intermediate and definitive hosts, including birds [11, 51].

Chemical pollutants add pressure through both direct and indirect pathways. Sublethal concentrations of pesticides, herbicides, and heavy metals can reduce cercarial survival, impair swimming behavior, and alter infectivity [52, 53]. Pollutants may also suppress snail immune responses, increasing susceptibility to trematode infection and parasite production [54, 55]. Contaminant exposure can either increase or decrease trematode prevalence depending on whether pollutants primarily suppress free-living parasite stages and intermediate hosts or weaken host defenses and simplify communities in ways that favor tolerant host–parasite combinations [56, 57].

Pollutants can also affect definitive avian hosts by modifying immune function and physiological condition. Heavy metals and organic pollutants can have immunosuppressive effects, including reduced lymphocyte activity and altered inflammatory responses [58, 59]. Experimental and comparative evidence suggests that compromised immune function may increase susceptibility to helminths and affect parasite establishment and persistence [60, 61]. Pollutant exposure may therefore increase trematode transmission to birds indirectly by weakening host defenses, not only by acting on parasites.

Eutrophication and pollution also interact with temperature and oxygen availability, producing complex transmission outcomes. Nutrient enrichment can intensify hypoxia through algal blooms and decomposition, reducing oxygen availability for snails and free-living parasite stages [51, 62]. Hypoxia reduces snail survival and cercarial emergence in some systems, although tolerant species may persist and dominate under low-oxygen conditions [63, 64]. Elevated temperature can amplify pollutant effects by increasing metabolic demand and toxicity, altering snail–trematode interactions and cercarial performance [31, 65]. These interactions indicate that trematode responses to environmental change are unlikely to be predicted reliably from single-stressor studies alone.

## Birds under climate change: shifting host availability and exposure

### Changes in bird distribution and range expansion

Many bird species, especially waterbirds and shorebirds tied to aquatic habitats, have shifted poleward and upslope in response to climate warming [66, 67]. Breeding and wintering areas of several duck, goose, and wader species show northward expansion across Europe and North America [68, 69], whereas elevational shifts in montane birds are creating novel high-elevation assemblages [70]. These changes alter the spatial availability of definitive hosts for trematodes across freshwater, coastal, and alpine systems [10, 30].

Range expansion also exposes birds to parasite communities with limited shared evolutionary or ecological history. Expanding populations can acquire local helminth faunas, including trematodes absent from their historical ranges [71, 72]. Newly established populations often show reduced parasite richness initially, followed by gradual accumulation of local species [73, 74]. Some trematodes recorded across expanded ranges may represent cryptic lineages rather than simple range extensions [27, 75].

Host range shifts can further create opportunities for host switching and parasite spillover. Trematodes may exploit novel definitive hosts when ecological overlap and trophic interactions permit transmission [12, 76]. In wetlands where resident and newly arriving bird species co-occur, infections in atypical hosts are consistent with ecological fitting, whereby parasites exploit novel hosts using preexisting traits such as compatible physiology, diet, or habitat overlap, rather than requiring new coevolutionary adaptation [77, 78]. Ecological fitting was also documented in periodically flooded oxbow lakes (e.g., Pansee) in the southern Czech Republic, where blackbirds feeding on dead fish were repeatedly infected with trematodes otherwise associated with fish-eating birds (J. Sitko, unpubl.). Genetic data indicate that some trematode populations infecting expanding bird hosts show little host-associated structuring, consistent with broad host competence [10, 79, 80]. These findings suggest that climate-driven range shifts can reorganize trematode transmission networks at regional scales through spillover and host switching.

### Phenological mismatches

Seasonal timing strongly structures trematode transmission because cercarial emergence, intermediate-host availability, and bird presence are all phenologically constrained [8, 24]. In many trematodes, cercarial emergence follows predictable seasonal patterns, mainly driven by temperature and photoperiod [23, 28]. At the same time, observational datasets show earlier spring migration and later autumn migration in waterbirds and shorebirds under climate warming [81, 82]. As a result, the temporal overlap between peak cercarial release and bird presence at stopover or breeding sites may either increase or decrease across latitudes. In some systems, warming can advance cercarial emergence and increase overlap with earlier-arriving or longer-staying birds. In contrast, in others, cercarial emergence may advance faster than bird migration or breeding phenology, reducing host–parasite encounter rates [20, 30].

Mismatches between bird migration stages and parasite transmission windows are already documented. Peak cercarial emergence in coastal and freshwater systems may precede or follow peak bird abundance, thereby reducing parasite–host encounter rates [8, 24]. Warm springs can advance cercarial emergence faster than bird arrival in some systems [20, 27]. Conversely, delayed migration has been linked to reduced trematode acquisition at traditional stopover sites [83]. Breeding phenology also shapes exposure risk, especially for chicks and juveniles. In waterfowl and shorebirds, hatching date influences both duration and intensity of exposure to infective stages in aquatic habitats [7, 8]. Early-hatched chicks may face greater exposure when cercarial densities are high, whereas later cohorts may encounter lower infection pressure as parasite output declines [13, 84]. Juveniles can also differ from adults in susceptibility and immune responses to helminths, affecting parasite establishment and development [60, 61].

These mismatches can have measurable effects on transmission success and parasite fitness. Reduced temporal overlap between cercarial emergence and the presence of the definitive host lowers parasite prevalence and intensity in bird populations [8, 24]. Field-based estimates further indicate that missed seasonal transmission windows can substantially reduce trematode lifetime fitness [44, 76]. Thus, climate-driven phenological shifts may reduce transmission for some species even where warming increases larval production, decoupling parasite performance from host availability and reshaping community composition. Empirical support for persistent phenological mismatch effects remains uneven across systems, and some bird–trematode assemblages may exhibit temporal buffering through extended migration windows, broad host ranges, or prolonged cercarial release periods.

### Behavioral and dietary shifts

Behavioral flexibility in foraging and habitat use strongly influences bird exposure to trematodes. Many trematodes infect birds through trophic transmission via metacercariae in aquatic invertebrates, fish, or amphibians [6, 12]. Climate- and resource-driven changes in prey composition can therefore alter exposure to second intermediate hosts [85, 86]. Birds shifting from benthic to pelagic prey, or from animal prey to more plant-based diets, show corresponding changes in trematode prevalence and intensity [87, 88].

Expansion of urban and agricultural landscapes has also promoted novel foraging strategies. Birds feeding in farmlands differ in trematode richness and infection dynamics compared with those in natural wetlands [88]. Long-term datasets indicate that agricultural habitats often support lower trematode diversity, with dominance by a few generalist species adapted to disturbance [88]. Similarly, urban foraging in ponds, canals, and drainage systems is associated with altered exposure owing to simplified intermediate-host communities and modified food webs [11, 41].

Artificial wetlands (reservoirs, fishponds, wastewater treatment plants, retention basins) are increasingly used by birds for feeding and resting. These systems often differ from natural wetlands in hydrology, nutrient loading, and intermediate-host composition, which can alter trematode transmission pathways [51, 90]. Loss and degradation of natural wetlands can restructure bird–trematode networks [91]. In particular, complex trematode assemblages associated with wetland birds may collapse under habitat homogenization and shifts in bird habitat use [91].

Behavioral shifts toward artificial or modified habitats can also destabilize transmission over time. Long-term monitoring of common birds using agricultural feeding habitats shows pronounced temporal fluctuations in trematode infections, likely reflecting variability in habitat management and prey availability [88]. In addition, reduced availability of suitable intermediate hosts in artificial wetlands can prevent completion of complex trematode life cycles even when birds remain abundant [91, 92]. Bird behavior and habitat choice can therefore decouple definitive-host presence from functional parasite transmission, contributing to regional declines in trematode diversity under ongoing environmental change.

## Trematode community restructuring in birds

### Changes in species richness and community composition

**Fig. 2:**
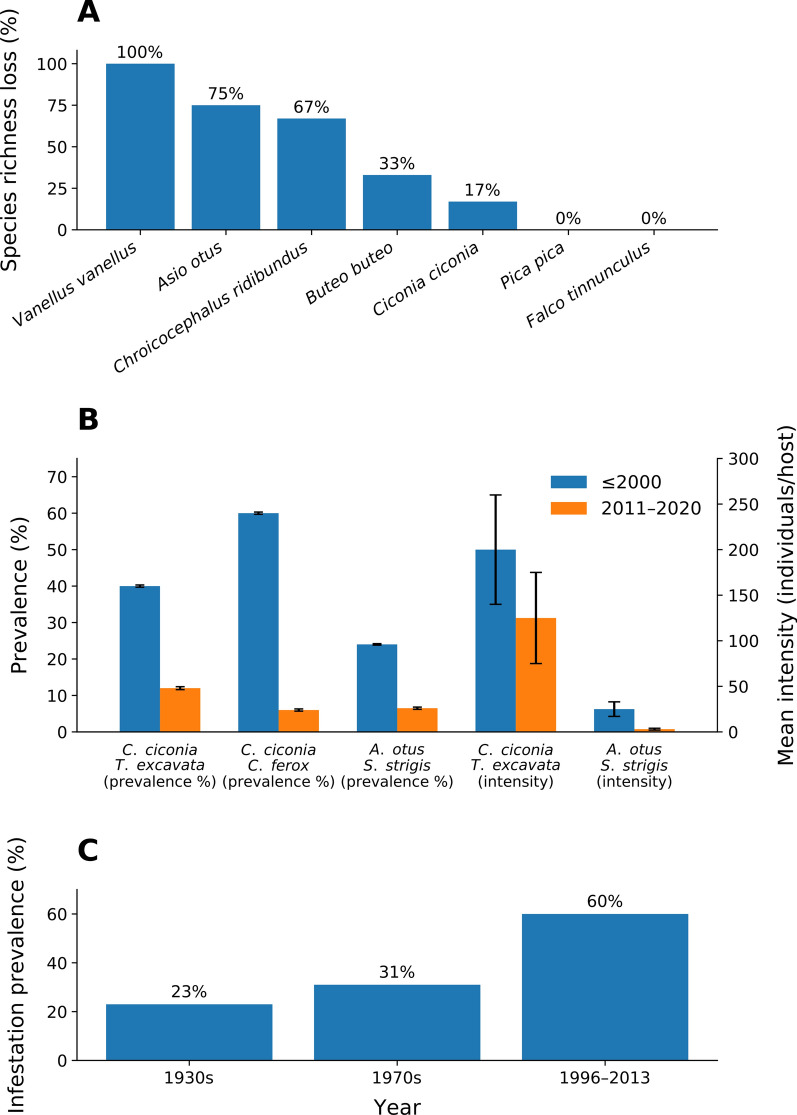
Long-term changes in bird trematode assemblages and infection metrics across time. **A** Species richness loss (%) in trematode assemblages recorded in individual bird host species, expressed relative to the earlier sampling period (set to 100%); values above the bars indicate percent loss for each host species shown [88, 91]. **B** Declines in infection prevalence (left y-axis; %) and mean intensity (right y-axis; individuals per host) of dominant trematodes in farmland-feeding birds, comparing historical records (> 2000) with recent surveys (2011–2020). The bars show the group values for each host–parasite pair as labeled; the error bars indicate the variability reported in the source data [88, 91]. **C** Increase in Notocotylidae infestation prevalence (%) in semiaquatic birds from Lake Chany (Western Siberia) across three survey intervals (1930s, 1970 s, and 1996–2013); the values above the bars represent prevalence for each period (23%, 31%, and 60%), indicating a marked long-term increase in infection occurrence [167]

Environmental change has been repeatedly associated with shifts in parasite richness and community composition across host taxa, including birds [30, 76]. Avian trematode assemblages are highly sensitive to habitat alteration, host availability, and disruption of multihost transmission pathways [6, 91] (Fig. 2). In anthropogenic landscapes, trematode richness in birds often declines even where host abundance remains high [11, 57].

Specialist trematodes appear disproportionately vulnerable. Trematodes with narrow host specificity or complex life cycles requiring multiple obligate intermediate hosts are more likely to disappear after wetland degradation, hydrological alteration, or loss of key hosts [18, 91]. By contrast, generalists that can exploit multiple definitive hosts or alternative intermediate hosts often persist or increase in disturbed systems [93, 94]. Long-term datasets from European wetlands document replacement of diverse, specialist-dominated assemblages by simplified communities dominated by widespread generalists [91]. This selective persistence promotes faunal homogenization.

Trematode communities in birds from anthropogenically modified wetlands are also becoming more similar across regions [27, 57]. Loss of regionally endemic or host-specific trematodes reduce beta diversity, paralleling patterns described in free-living aquatic taxa [95, 96]. Molecular surveys further suggest that apparently stable species lists may mask lineage replacement by broadly distributed taxa, reinforcing functional homogenization [75, 97, 98]. Community restructuring patterns vary by climatic region. In temperate systems, substantial declines in trematode diversity are linked to wetland loss, agricultural intensification, and altered bird habitat use [90, 91]. Tropical systems may retain greater trematode diversity where greater host diversity and year-round transmission support complex life cycles, although localized losses occur after deforestation and eutrophication [99]. High-latitude systems, once considered parasite-poor, are yielding increasing trematode records as surveillance improves; warming and shifting bird communities may promote faunal interchange and novel host–parasite associations [100–102]. Thus, environmental change can drive both gains and losses in trematode diversity depending on baseline community structure, host availability, and climatic context.

### Emergence of novel host–parasite associations

Recent decades have produced increasing first records of trematodes in avian hosts and regions where they were previously unreported (Table 1). In some cases, these detections involve well-studied bird species, suggesting actual emergence rather than only historical undersampling [91, 100]. Environmental change can facilitate such associations by altering contact rates among birds, intermediate hosts, and parasite transmission stages [10, 30].

**Table 1:** Shifts in bird distribution (poleward or sub-Arctic occurrence) associated with changes in trematode assemblages or novel host–parasite records

**Bird host context**	**Region and time window**	**Trematode (life-cycle context)**	**Quantitative bird or trematode evidence**	**Evidence type**	**Sources**
Increased habitats and numbers of fish-eating birds	Volga basin, European Russia; change assessed over 87 years	*Clinostomum complanatum*	The northern distribution limit has moved approximately 100 km north	Range-limit shift of a bird-transmitted trematode	Zhokhov and Pugacheva [163]
Human cercarial dermatitis outbreaks in an urban pond system	Reykjavík, Iceland; late summers 1995–1997	Schistosome cercariae	Late summer outbreaks documented for 1995–1997; described as the first report in Iceland and the most northern occurrence in Europe	New regional record (poleward direction)	Kolářová et al. [164]
Sub-Arctic extension and first Icelandic record of a raptor trematode	Iceland; 2024	*Strigea falconis* (Iceland is thought to lack suitable intermediate hosts for completion of its life cycle)	Reported as the first record from Iceland; the Icelandic gyrfalcon population is sedentary	Novel regional record in a definitive host at high latitude	Faltýnková et al. [165]

Migratory connectivity is central to establishing new host–parasite links. Migratory birds connect distant wetlands and coastal systems and can transport parasites beyond historical ranges [9, 10]. Stopover sites with high bird turnover may harbor trematode assemblages of mixed biogeographic origin [103]. Identical or closely related trematode lineages occurring in birds across distant migration-linked regions support long-distance dispersal mediated by avian hosts [27, 75]. Novel associations arise through multiple mechanisms. Many trematodes appear to have broad physiological compatibility with avian definitive hosts, allowing establishment in novel hosts without prior coevolutionary history [12, 76]. Cases involving atypical bird hosts often reflect dietary and habitat overlaps rather than close host phylogenetic relatedness [6, 104, 105]. This pattern is consistent with ecological fitting, in which parasites exploit preexisting traits when new opportunities arise [78].

Evidence for rapid evolutionary adaptation after host switching remains limited in bird trematodes. Trematode population structure is often shaped more by dispersal through the most mobile host(s) than by host-associated subdivision; parasites using birds or mammals as definitive hosts frequently show reduced genetic structure, consistent with ongoing gene flow [80, 106, 107]. Host expansion can therefore be facilitated by ecological fitting rather than novel adaptation [108–110]. Most newly observed trematode–bird associations are thus best explained by ecological opportunity, with environmental change reshaping contact networks and increasing encounter frequency.

### Cryptic diversity and hidden turnover

Molecular systematics has shown that morphologically defined trematode taxa often comprise multiple cryptic species with distinct host associations and ecological requirements. In freshwater and marine systems, deep genetic structuring is common among nominal bird-infecting trematodes and their intermediate host stages [27, 111]. In several cases, cryptic lineages differ in their geographic distributions and host use, suggesting distinct responses to environmental conditions despite similar morphology [112, 113]. Environmental change may therefore affect cryptic species unevenly, even when the prevalence of a morphospecies appears stable.

Evidence from complex life-cycle taxa further indicates that cryptic species can differ in sensitivity to temperature, hydrology, and host availability. Molecular analyses of *Tylodelphys* spp. revealed multiple cryptic species with distinct transmission pathways and ecological niches despite morphological similarity [89]. Experimental and field observations suggest corresponding differences in intermediate host use and habitat preferences, implying divergent responses to environmental change [89]. Comparable patterns are reported in avian Dicrocoeliidae, where molecular data uncover previously unrecognized species boundaries and host specificity [114]. Reliance on morphology alone can thus misrepresent community stability: replacement of genetically distinct lineages may mask local extinctions, colonizations, or host-use shifts detectable only with molecular tools.

These issues directly affect the interpretation of long-term parasitological records. Historical datasets underpin many assessments of environmental-change effects on parasite communities, but most lack molecular resolution [6, 76]. Reanalysis of archived material with genetic methods has revised species lists and altered conclusions about temporal trends in parasite diversity [114]. Assessments based only on morphology therefore risk underestimating community turnover and overestimating parasite persistence. Integrating molecular identification into long-term monitoring is essential for accurately tracking how trematode communities in birds restructure in response to ongoing environmental change.

## Birds as amplifiers, buffers, or breakpoints in changing systems

Birds sit at pivotal points in trematode life cycles and can act as amplifiers, buffers, or transmission breakpoints, depending on ecological context. Because mobility, abundance, and trophic interactions shape encounter rates and dispersal, shifts in bird communities can determine whether trematode transmission persists, expands, or collapses under environmental change [12, 76]. Consequently, community-level changes can drive system-wide trematode dynamics beyond individual host–parasite interactions.

### Birds as dispersal agents of trematodes across landscapes

Bird movement facilitates long-distance transport of trematodes among aquatic systems. Identical trematode lineages can infect birds across distant wetlands linked by migratory flyways [9, 10]. Eggs and other propagules shed by birds may remain viable after long-distance movement and contribute to establishment where suitable intermediate hosts occur [12, 71]. Wetlands used by migratory birds, therefore, often support higher trematode richness than isolated sites with limited bird connectivity [103, 115].

Dispersal can promote both persistence and spatial reorganization of trematode populations. Introduction into newly suitable habitats has been documented following climate-driven changes in bird ranges and stopover use [100, 102]. Frequent movement among sites may also homogenize trematode assemblages by maintaining gene flow across populations [27]. Birds thus link local trematode dynamics to regional and continental processes.

### Dilution versus amplification effects in altered communities

Changes in bird community composition can modify trematode transmission through dilution or amplification effects. Higher diversity of definitive hosts can reduce transmission efficiency for some parasites by lowering encounters with competent hosts [11, 116]. Conversely, dominance by highly competent bird hosts is associated with increased prevalence and intensity in intermediate hosts [8, 74]. Habitat simplification commonly favors generalist bird species with high contact rates with infected prey, promoting amplification of trematode transmission [91].

Whether dilution or amplification occurs depends on competence, abundance, and behavioral overlap with transmission stages. It also depends on the environmental setting in which cercariae are produced and encounter intermediate hosts; for example, warming and low-tide conditions can strongly increase emergence in intertidal trematodes, thereby altering the background level of transmission opportunity independent of bird-community composition [117]. Loss of rare or specialist birds can reduce host diversity without reducing transmission, yielding net amplification by remaining hosts [88, 116]. In contrast, increases in bird diversity following habitat restoration have been linked to reduced infection pressure in some systems [4]. Biodiversity change does not produce uniform outcomes; it reshapes host–parasite interactions in strongly context-dependent ways. These outcomes are not contradictory but describe different consequences of bird-community change. Dilution and amplification primarily refer to changes in infection pressure or transmission intensity for particular trematode taxa, whereas the loss of key bird hosts affects the persistence of whole life cycles and, therefore, parasite richness. Thus, the same process of community simplification can increase the abundance or prevalence of trematodes that use abundant, competent generalist birds, while simultaneously reducing the diversity of specialist trematodes that require declining or behaviorally displaced host species. The following section, therefore, considers the complementary case in which altered bird communities serve as breakpoints in trematode life cycles rather than amplifiers of transmission.

### Loss of key bird species and cascading effects on trematode life cycles

Whereas community simplification may amplify transmission by concentrating exposure in abundant and competent generalist hosts, it can have the opposite effect on trematode diversity when the lost species are required definitive hosts. Some bird species function as key definitive hosts required for the completion of complex trematode life cycles. Their decline or removal can interrupt transmission even when intermediate hosts remain abundant [8, 13]. Long-term monitoring in European wetlands shows that declines in specialist wetland birds coincide with the disappearance of trematodes that depend on those hosts [91]. Experimental exclusion of birds from intertidal systems also led to rapid declines in trematode prevalence in snails and other intermediate hosts [118].

Loss of key hosts can trigger cascading effects across parasite communities. Trematodes with complex life cycles are particularly vulnerable when definitive hosts decline or shift habitat use [18]. As bird communities simplify, trematode diversity can decline disproportionately relative to host loss [91]. In this way, birds can act as breakpoints in trematode transmission networks, where changes in a few key hosts produce systemic collapse rather than gradual decline.

## Ecosystem-specific contexts of bird–trematode transmission

The following section compares how bird–trematode transmission is reorganized across major ecosystem types. We focus here on ecosystem-specific contexts in which the same broad drivers, namely temperature, hydrology, habitat structure, intermediate-host availability, and bird movement, produce different transmission outcomes. We highlight where freshwater, coastal, and high-latitude systems differ mechanistically.

### Freshwater systems

Freshwater ecosystems are among the best-studied settings for trematode transmission involving birds. Lakes, rivers, and reservoirs support diverse intermediate snail hosts, aquatic prey, and avian definitive hosts, producing tightly coupled transmission networks [11, 36]. Trematode dynamics in these systems can respond quickly to climatic variability, hydrological change, and shifts in bird habitat use [30, 90].

In lakes, temperature-driven changes in stratification and productivity influence snail dynamics and cercarial production. Warmer summers can increase snail growth and extend the seasonal transmission window in many temperate lakes [20, 22]. Higher cercarial output can increase infection pressure on second intermediate hosts and waterbirds foraging in littoral habitats [8, 51]. By contrast, episodic hypoxia associated with eutrophication and warming can suppress sensitive snail species and shift trematode community composition [111].

River systems often exhibit pronounced spatial heterogeneity in transmission, driven by flow regime and connectivity. Slow-flowing sections and impounded reaches typically support higher snail densities and trematode prevalence than fast-flowing segments [119, 120]. By exploiting regulated rivers and floodplains, birds maintain transmission across hydrologically distinct reaches [10]. Flood events can temporarily increase connectivity and transmission, often followed by declines during high-discharge periods [37, 38, 121].

Reservoirs, increasingly common freshwater habitats, can support distinct trematode dynamics. They often favor generalist snails and bird hosts adapted to stable water levels, producing simpler assemblages than those in many natural lakes [41, 91]. High bird abundance may still sustain intense transmission for a subset of trematodes with flexible life cycles [90]. Thus, reservoirs can function as focal points for both persistence and restructuring of trematode communities.

Climate-driven feedbacks among snails, birds, and trematodes are increasingly evident. Warming can increase snail productivity and advance the arrival of birds at breeding or stopover sites, thereby reinforcing transmission when conditions align [20, 69]. Conversely, mismatches between snail infection periods and bird presence can suppress transmission even when larval output is high [8]. Freshwater systems can therefore shift rapidly between amplification and collapse as climatic and biological drivers interact.

### Coastal and marine systems

Coastal and marine ecosystems differ from freshwater systems mainly in the strong spatial coupling between intertidal habitat, molluscan or crustacean intermediate hosts, and foraging shorebirds or seabirds. Rocky shores, mudflats, and salt marshes can support dense assemblages of intermediate hosts and high trematode transmission, but exposure is tightly constrained by tidal cycles, temperature, and the timing of bird use [8, 118]. Thus, coastal transmission often depends less on basin-scale hydrology than on the persistence of intertidal foraging habitat and the synchrony between cercarial emergence, invertebrate host availability, and bird migration.

Sea-level rise and coastal modification are emerging as key drivers. Intertidal habitat loss from coastal squeezing and shoreline hardening reduces habitat for intermediate hosts and foraging birds [102, 122]. Reduced intertidal area can lower trematode prevalence by disrupting host contact even where birds remain abundant [8, 118]. Fragmentation can concentrate hosts and birds into smaller areas, thereby locally increasing transmission intensity [122].

Marine trematode communities also respond to changes in host populations and behavior. Overharvesting, invasions, and disease-driven declines in key invertebrate hosts can reduce trematode richness and alter community composition [73, 123]. Changes in migration routes and stopover duration modify the temporal availability of definitive hosts, shifting transmission windows in estuaries and intertidal habitats [10, 24]. Coastal assemblages frequently include cryptic species with distinct host-use patterns, complicating interpretation of apparent stability on the basis of morphology alone [113, 124].

Human activities further shape the dynamics of coastal trematodes. Nutrient enrichment, pollution, and aquaculture can alter invertebrate communities and either increase or suppress transmission depending on local conditions [56, 57]. Protected coasts with more intact food webs often support higher trematode diversity than heavily modified areas [8, 118]. Coastal trematode systems can reorganize rapidly under interacting climatic and anthropogenic pressures, with birds mediating both persistence and breakdown of transmission networks.

### High-latitude and high-elevation systems

High-latitude and high-elevation aquatic systems have short ice-free seasons and strong seasonality that constrain the completion of trematode life cycles linked to birds [2]. Evidence from a subarctic lake shows that a bird schistosome can sustain relatively high cercarial output at low temperatures, with emergence following a circadian rhythm aligned with the day-night cycle [125] (Table 2). In Arctic intertidal habitats of the Pechora Sea region, trematode assemblages in *Littorina saxatilis* include microphallids and other digeneans whose transmission persists despite harsh conditions, likely because second intermediate hosts and seabird final hosts remain available [126]. Over decades at this range edge, increases in microphallid prevalence and apparent range expansion of some taxa have been reported, with climate warming and changing bird distributions proposed as drivers [126].

**Table 2:** Subarctic bird schistosome cercarial emergence under low-temperature conditions

**Experimental context**	**Water temperature (mean; range, °C)**	**Output metric reported**	**Outcomes**
Field, October 2018	6.4 (5.9–6.8)	Pooled daily output rates	Total = 38 cercariae (from initial snails); range = 0–12; mean ± SD = 2 ± 4 cercariae/snail/day
Laboratory, October 2018	5.7 (5.5–6.5)	Pooled daily output rates	Total = 21 cercariae; range = 0–21; mean ± SD = 1 ± 5 cercariae/snail/day
Field, August 2016	8.3 (6.6–9.9)	Pooled daily output rates	Total = 2101; range = 1–1295; mean ± SD = 150 ± 337 cercariae/snail/day
Field, August 2016 (diel emergence totals by interval; day 1)	8.3 (6.6–9.9)	Observed total (mean) by diel interval	0:00–6:00 = 1704 (243); 6:00–12:00 = 131 (19); 12:00–18:00 = 114 (16); 18:00–0:00 = 20 (3)
Infection prevalence in snails	6.4 (5.9–6.8)	Patent and prepatent prevalence	October 2018: 14/689 (2.0%) infected with patent and prepatent *Trichobilharzia*; eight with patent infections

All data in this table concern *Trichobilharzia* sp. “peregra” in *Radix balthica* from Lake Takvatn (69°07’N, 19°05’E, Northern Norway), as published by Soldánová et al. [125]

High-latitude freshwater systems can also support substantial trematode diversity in snails, consistent with bird-mediated dispersal along flyways [2]. A multi-regional high-latitude survey of echinostomes documented diverse lineages in snails from Iceland, Finland, Ireland, and Alaska, highlighting migratory birds as frequent definitive hosts connecting the parasite pools [127]. In Iceland, Pantoja et al. similarly emphasized the island as a significant nesting and staging area on the East Atlantic Flyway, supporting repeated trematode introduction and persistence in northern lakes [127].

Disease-risk models integrating long-term wildlife datasets predict larger increases in parasite prevalence at higher latitudes and elevations under warming than at lower latitudes [128]. Lengthening transmission seasons in alpine and Arctic wetlands may increase the number of successful trematode cohorts per year for bird-associated taxa where development and emergence are presently season-limited [2, 128], consistent with mechanistic expectations linking warming to elevated disease risk [129]. High-elevation systems may therefore function as sentinel landscapes where modest thermal shifts yield significant proportional increases in time suitable for snail activity and parasite development [2, 128]. Upslope or poleward advances of competent snails, combined with changes in waterbird use of high-elevation lakes, are probable mechanisms for the rapid emergence of sustained trematode transmission at sites that historically lacked cycling [2].

## Implications for avian health, population dynamics, and conservation

### Sublethal effects under environmental stress

Experimental infection with the oviduct trematode *Prosthogonimus macrorchis* reduced egg production in ring-necked pheasants, demonstrating a direct reproductive cost of a bird trematode [130]. Field data from tufted ducks (*Aythya fuligula*) linked trematode burdens to variation in body condition, suggesting that infection intensity can covary with energetic state in free-ranging birds [131]. Controlled infections of domestic ducks elicited measurable humoral responses to the avian schistosome *Trichobilharzia regenti*, indicating activation of avian immune pathways [132]. Experimental work on *T. regenti* further demonstrated neurotropic migration routes in ducks, providing mechanistic pathways for pathology that can occur before mortality end points [133]. In small-scale duck production systems, helminth infections (including trematodes) are associated with reduced weight gain and egg production, supporting the premise that even nonlethal helminthiasis can impose performance costs under resource limitation [134]. Environmental stressors that reduce foraging efficiency or increase energetic demands are therefore expected to increase the likelihood that sublethal trematode effects translate into demographic costs [135].

### Parasites as hidden contributors to population declines

Epizootic mortality of migrating waterbirds linked to intestinal trematodes has been documented repeatedly since 2002 in the Upper Mississippi River National Wildlife and Fish Refuge system [136, 137]. In the Upper Mississippi River system, migration bottlenecks concentrate large numbers of waterbirds at stopover sites where suitable foraging habitats are spatially restricted. Species such as lesser scaup and American coot forage intensively on benthic prey within these areas, leading to repeated ingestion of infective stages associated with intermediate hosts, including the invasive snail *Bithynia tentaculata*. This combination of high host density and focused foraging activity amplifies exposure rates, leading to exceptionally high parasite burdens and, in some cases, mortality. Thus, the bottleneck effect emerges not only from spatial aggregation of hosts but from the coupling of aggregation with trophic transmission pathways during concentrated feeding. Infection patterns in such systems vary among co-occurring bird species owing to differences in diet composition, foraging behavior, and habitat use, which determine exposure to infected intermediate hosts [137]. Spatial and temporal variation in intermediate-host abundance and infection prevalence further modulates exposure risk, producing marked heterogeneity in parasite burdens across sites and seasons [138]. Under conditions of high host aggregation, particularly during migration or prebreeding periods, these processes can lead to exceptionally high parasite loads and associated mortality, with potential carryover effects on subsequent reproduction [135, 137].

### Parasite loss versus parasite overload in disturbed systems

Long-term surveillance of wetland-associated birds in Europe revealed systemic collapse of a trematode network, indicating that disturbance and ecosystem reconfiguration can drive sustained parasite loss at the community level [91]. A complementary long-term dataset from farmland-associated feeding habitats found marked declines in prevalence and intensity across multiple trematode species in common bird hosts, suggesting that parasite loss can extend across functional habitats used by birds [88]. By contrast, invasive host-mediated systems in North America have produced recurrent trematode-associated die-offs, illustrating a parasite-overload regime that can emerge following community reassembly and the establishment of novel intermediate hosts [136–138]. Conservation planning in altered landscapes, therefore, needs to consider both directions of change, because parasite loss and parasite overload can co-occur across regions and seasons within the same flyway [91, 137].

### Relevance for endangered and migratory bird conservation

Because trematode transmission depends on aquatic intermediate hosts, conservation actions that alter wetland hydrology and connectivity can change exposure risk for migratory birds congregating at stopover sites [138]. The Upper Mississippi River case study illustrates how stopover aggregation, invasive snails, and concentrated benthic foraging by birds can align to generate high infection pressure, making it a useful model for risk assessment at migration bottlenecks [136, 137]. Evidence for long-term trematode community decline in European wetland and farmland birds also suggests that parasite and host conservation can intersect, as parasite loss may accompany broader simplification of food webs and habitat function [88, 91]. For threatened migratory species, trematode management is likely to be most effective when integrated with site-based actions that jointly address intermediate host habitats, bird aggregation patterns, and exposure timing [137, 138].

## Human relevance: avian schistosomes and cercarial dermatitis under climate change

Avian schistosomes provide the clearest direct link between bird trematode ecology and human health. Unlike human schistosomes, avian schistosomes do not complete development in humans; people are accidental, nonpermissive hosts. However, cercariae released from infected snails can penetrate human skin and cause cercarial dermatitis, also known as swimmer’s itch. Thus, although these parasites do not cause human schistosomiasis in the strict sense, they represent a human-relevant outcome of bird–snail trematode transmission.

Climate change may increase the frequency, duration, and geographic extent of human exposure to avian schistosome cercariae through several interacting pathways. Warmer water can accelerate intramolluscan development, increase cercarial production, and extend the seasonal window during which infected snails release cercariae [19, 20, 90]. At the same time, shifts in waterbird migration, overwintering, and breeding distributions can alter the local availability of definitive hosts that maintain schistosome transmission [66, 69]. Where warming promotes longer residence of infected or competent bird hosts at recreational lakes, ponds, and reservoirs, parasite egg input into snail habitats may increase.

Hydrological alteration and eutrophication may further amplify risk. Nutrient enrichment can increase snail abundance by enhancing primary productivity and food availability, while stable or slow-flowing waters often favor snail hosts and human recreational use [36, 47, 51]. Artificial lakes, urban ponds, reservoirs, and bathing sites can therefore become transmission hotspots when suitable snails, infected waterbirds, and human bathers overlap spatially and seasonally. Reports of cercarial dermatitis outbreaks in northern and urban water bodies illustrate how avian schistosome transmission can emerge in settings where climate, bird use, snail habitat, and human exposure converge [164].

The public health relevance of avian schistosomes is therefore expected to be highly context-dependent. Warming alone is unlikely to produce uniform increases in swimmer’s itch, because cercarial survival, snail host suitability, bird abundance, and human water contact may respond differently across systems. In some habitats, extreme heat, drought, or hydrological disturbance may suppress snail populations or shorten cercarial survival, reducing exposure. In others, moderate warming combined with eutrophication, stable water levels, and abundant waterbirds may increase cercarial output and prolong the bathing-season risk window. Therefore, predicting human exposure requires integrated surveillance of waterbirds, snail hosts, cercarial emergence, and recreational water use rather than monitoring temperature alone.

## Methodological challenges and knowledge gaps

### Sampling biases

Most knowledge of avian trematodes comes from opportunistic necropsies (hunting, ringing mortality, or rehabilitation centers), producing nonrandom sampling of host species, ages, and health states [139, 140]. Long-lived and protected species are underrepresented because lethal sampling is often restricted, limiting inference about lifetime accumulation and chronic infections [32, 141]. Seasonal bias is also common because sampling clusters in hunting seasons or breeding periods, reducing sensitivity to migration- or hydrology-linked variation in transmission [2, 20]. Geographic coverage remains uneven, with Europe and parts of East Asia overrepresented relative to Africa, South America, and much of the tropics where diversity may be highest [96]. These biases can obscure large-scale responses to environmental change and create misleading impressions of stability or decline.

### Limits of classical taxonomy

Morphology-based identification has dominated avian trematode research, but many taxa are morphologically conservative and can mask substantial genetic divergence [142]. Cryptic species complexes are repeatedly uncovered within nominal bird-infecting taxa, including lineages with distinct host use and ecological preferences [89, 114]. Reliance on morphology alone can therefore underestimate species richness and misrepresent host specificity and geographic ranges [143]. Long-term datasets based only on morphology may suggest persistence even where lineage turnover has occurred at the genetic level [114]. Integrative taxonomy, combining morphology, molecular data, and ecological information, is increasingly essential for detecting biologically meaningful changes in avian trematode communities.

### Scaling from individuals to flyways

Most studies operate at local or regional scales and focus on individual wetlands or host populations, limiting their ability to link infection patterns to processes across migratory flyways [144, 145]. Migratory behavior can connect distant parasite populations via shared definitive hosts, but integrated datasets combining parasite genetics, bird movements, and intermediate-host distributions remain scarce [146]. Differences in sampling effort and methods across regions further complicate comparisons [140]. Coordinated programs integrating parasitology with banding, telemetry, and molecular surveillance are still uncommon [145, 147]. Without explicit scaling from hosts to flyways, predictions about trematode responses to global environmental change remain largely inferential.

### Limitations

The limitations outlined above mean that several conclusions in this review should be interpreted as evidence-based hypotheses rather than definitive causal relationships. In many cases, observed changes in trematode transmission or community composition are associated with environmental change, altered host distributions, or habitat modification, but the relative contributions of these drivers cannot be separated with confidence. Laboratory studies provide valuable mechanistic insight into thermal sensitivity, but their results may not fully capture how temperature interacts with hydrology, eutrophication, pollutants, host behavior, and community turnover under field conditions. Therefore, extrapolations across ecosystems, host assemblages, and climate scenarios should be made cautiously and tested through coordinated longitudinal studies that combine parasite identification, host movement data, and environmental monitoring.

## Future directions

### The use of bird trematodes as early warning indicators of environmental change

Trematodes with complex life cycles can act as sensitive indicators because successful transmission requires alignment of multiple hosts and free-living stages. Trematode richness and prevalence in birds can decline rapidly after wetland degradation, eutrophication, or hydrological simplification, sometimes preceding detectable changes in bird community structure [91, 148]. Long-term European datasets show that the collapse of bird-associated trematode networks coincides with the loss of intermediate-host diversity and altered water regimes [91]. Comparative work suggests parasite assemblages integrate information across aquatic and terrestrial components, making them useful sentinels of ecosystem connectivity [49, 144]. Relative to host metrics alone, trematode assemblages in birds may therefore provide earlier and more integrative signals of ecosystem disruption.

### Integrating parasitology with movement ecology and remote sensing

Advances in movement ecology reveal fine-scale patterns of habitat use, stopover fidelity, and migratory connectivity relevant to parasite exposure and dispersal [149, 150]. Tools such as stable isotopes and GPS tracking can link parasite burdens to foraging habitats and migratory routes [145]. Remote sensing products describing surface-water dynamics, wetland phenology, and temperature anomalies provide spatial predictors of trematode transmission windows at large scales [151]. Integrating parasite sampling with movement and environmental data has improved the prediction of infection risk in waterbirds across heterogeneous landscapes [152–154]. A unified framework combining parasite data, bird movement, and remotely sensed environmental variables could shift trematodes from retrospective indicators to predictive tools.

### Predictive models of parasite redistribution under climate change scenarios

Mechanistic and correlative models project poleward and elevational shifts in trematode transmission driven by temperature-dependent development and changing host distributions [155, 156]. However, warming can accelerate development while increasing mortality beyond thermal optima, producing nonlinear responses that complicate extrapolation [20, 22]. Coupled host–parasite models incorporating bird migration indicate that definitive-host mobility can either facilitate rapid range expansion or buffer local extinction through recolonization [157–159]. Despite their relevance, scenario-based projections that integrate climate models with host movement remain rare for avian trematodes. Predictive modeling of redistribution under climate change is therefore underdeveloped but tractable. These projections remain constrained by limited long-term datasets, inconsistent sampling intensity among regions, and uncertainty regarding how host behavior, hydrological variability, and community turnover interact under future climate scenarios.

### Key testable hypotheses for the next decade

Comparative field studies along latitudinal gradients can test whether trematode assemblages in birds shift from specialist- to generalist-dominated communities as environmental variability increases [91, 160]. Longitudinal monitoring of migrants can evaluate whether altered migration timing produces consistent mismatches with peak cercarial release and reduced transmission success. Integrative taxonomic approaches combining morphology with multilocus sequencing can test whether cryptic lineages differ in environmental tolerance and host use [89, 114]. These tests can distinguish ecological filtering, evolutionary responses, and loss of transmission opportunities as drivers of observed change.

## Conclusions

Environmental change is reshaping bird–trematode systems through interactions among climate, hydrology, habitat structure, and host behavior. Available evidence suggests that trematode assemblages in birds can respond rapidly to environmental pressures and may, in some systems, reveal ecological disruption before changes become apparent at higher trophic levels. Birds likely influence trematode persistence, dispersal, and local extinction dynamics across landscapes through their mobility and trophic interactions. Understanding these dynamics is essential for advancing parasitology, interpreting long-term changes in biodiversity, and integrating parasites into avian ecology and conservation science. Future progress will depend on integrative and longitudinal approaches that combine parasitology, movement ecology, molecular identification, and environmental monitoring across broader geographic scales.

## Data Availability

No datasets were generated or analyzed during the current study.
